# Silencing of *Entamoeba histolytica* Glucosamine 6-Phosphate Isomerase by RNA Interference Inhibits the Formation of Cyst-Like Structures

**DOI:** 10.1155/2013/758341

**Published:** 2013-01-22

**Authors:** Hugo Aguilar-Díaz, Juan Pedro Laclette, Julio César Carrero

**Affiliations:** Departamento de Inmunología, Instituto de Investigaciones Biomédicas, Universidad Nacional Autónoma de México, A.P. 70228, Mexico City, DF, Mexico

## Abstract

Encystment is an essential process in the biological cycle of the human parasite *Entamoeba histolytica*. In the present study, we evaluated the participation of *E. histolytica* Gln6Pi in the formation of amoeba cyst-like structures by RNA interference assay. Amoeba trophozoites transfected with two Gln6Pi siRNAs reduced the expression of the enzyme in 85%, which was confirmed by western blot using an anti-Gln6Pi antibody. The *E. histolytica* Gln6Pi knockdown with the mix of both siRNAs resulted in the loss of its capacity to form cyst-like structures (CLSs) and develop a chitin wall under hydrogen peroxide treatment, as evidenced by absence of both resistance to detergent treatment and calcofluor staining. Thus, only 5% of treated trophozoites were converted to CLS, from which only 15% were calcofluor stained. These results represent an advance in the understanding of chitin biosynthesis in *E. histolytica* and provide insight into the encystment process in this parasite, which could allow for the developing of new control strategies for this parasite.

## 1. Introduction

The intestinal protozoan parasite *Entamoeba histolytica,* the causal agent of human amoebiasis around the word, is considered a serious problem of public health mainly in developing countries. Amoebiasis is a major source of morbidity and mortality with estimate of 50 million people infected annually [1]. The *E. histolytica* life cycle includes two biological forms: the trophozoite and the cyst. The cyst is the infective form and has a tetranucleated structure surrounded by a chitin wall highly resistant to adverse environment conditions [2]. In spite of numerous *in vitro* studies focused on obtaining the infective mature forms of *E. histolytica* cysts, no reproducible procedure has yet been developed. However, a recent study by our group achieved induction of cyst-like structures (CLS) with a characteristic chitin thick wall, refringency, multinucleation (even a few of CLS with 4 nuclei) and chromatoid bodies by treatment of trophozoites with hydrogen peroxide in the presence of trace amounts of several metallic dications [3]. The CLS obtained was resistant to lysis by detergents and exposition to environmental conditions for several weeks (unpublished data). These results suggest that encystment pathways can be activated in *E. histolytica* trophozoites when they are exposed to oxidative stress, mainly hydrogen peroxide, as it has been suggested for differentiation in other cells (reviewed in [4]). In this regard, exposure to luminal conditions, such as reactive oxygen species from immune cells or intestinal microbiota, has been suggested to be involved in amoeba encystment [5, 6]. In addition, a role for certain divalent cations as cofactors of enzymes involved in excystment and encystment has also been described [7]. However, the cellular events behind the synthesis of the chitin wall and, in general, the developmental conversion of *E. histolytica*, are still unknown. 

The *in vitro* encystment of other protozoans such as *Giardia lamblia* and *E. invadens* is extensively studied (reviewed in [8]). During the encystment of* G. lamblia*, it has been observed that the filamentous structure of the cyst wall consists of a polymer of *N*-acetylgalactosamine [9]. This polymer is synthesized from endogenous glucose by a biosynthetic pathway where the rate-limiting enzyme is glucosamine-6-phosphate isomerase (Gln6Pi), an enzyme that reversibly isomerizes fructose-6-phosphate to glucosamine-6-phosphate [10]. Accordingly, we also reported that *in vitro* induction of *E. histolytica* CLS is coupled to overexpression of a Gln6Pi, as determined by RT-PCR [3]. However, solid evidence of the functional participation of this enzyme in amoeba encystment was not provided.

RNA interference (RNAi) method is a powerful tool for assessing and manipulating gene function. In this process, a double-stranded RNA (dsRNA) can initiate posttranscriptional sequence-specific silencing of cellular genes by mRNA degradation [11]. The mechanism of RNAi may have evolved as a defense against viruses and transposable elements with dsRNA intermediates [12, 13]. In this process, the small RNA intermediates, short interfering RNAs (siRNAs), result from dsRNA being cleaved at 21 to 23 nucleotide intervals [14] by RNase III-type [15] and are then incorporated into the RNA-induced silencing complex (RISC) and guided to their target mRNA, which is then cleaved by Argonaute proteins [14, 16]. So far we know that the molecular RNAi machinery and siRNAs are present in *E. histolytica* and the silencing of genes in this parasite is relatively common (reviewed in [17, 18]).

In this work, we evaluated the participation of Gln6Pi in the formation of the CLS by RNA interference assay. Our results show that under encystment induction treatment, the Gln6Pi knockdown in trophozoites results in the loss of the capacity of trophozoites to synthesize the chitin wall, and therefore, to transform into a CLS, suggesting a pivotal role for Gln6Pi in *E. histolytica* encystment.

## 2. Material and Methods

### 2.1. Parasite Cultures

*E. histolytica *trophozoites, HM-1: IMSS strain, were grown at 37°C in sterile TYI-S-33 medium supplemented with 10% adult bovine serum, 100 U/mL of penicillin, 100 mg/mL of streptomycin sulfate, and 3% of Diamond Vitamins [19]. 

### 2.2. Induction of Cyst-Like Structures

The induction of cyst-like structures (CLS) was performed as previously reported [3]. In brief, trophozoites were chilled on ice for 5 min and harvested by centrifugation at 150 ×g for 7 min at 4°C. The cells obtained (1 × 10^5^) were resuspended in 50 mL of fresh TYI-S-33 media in culture flasks and incubated at 37°C. After 72 h, trophozoites in log phase (approximately 5 × 10^6^ cells) were treated with 4 mM of a 30%  H_2_O_2_ solution containing different traces of several dications (cadmium 0.02 ppm, cobalt 0.02 ppm, copper 0.02 ppm, iron 0.1 ppm, nickel 0.02 ppm, lead 0.02 ppm, zinc 0.02 ppm) and other components (free sulfuric acid 40 ppm, chlorine 0.5 ppm, phosphate 5 ppm, and sulfate 2 ppm) (Merck UN 2014, Darmstadt, Germany). The cultures were then incubated at 37°C for 6 h. After treatment, parasites were washed two times with phosphate buffered saline, counted under microscope and resuspended in PBS containing 0.5% sarkosyl, and allowed to sit for 10 min at room temperature. After three washes as above, the detergent-resistant trophozoites were resuspended in PBS and counted again in a microscope. The conversion rate index was estimated as the percentage of parasites that were resistant to sarkosyl and therefore were converted from trophozoites to CLS. Three independent experiments by triplicate were carried out for each analysis.

### 2.3. Small Interference RNA (siRNA)

Design of siRNAs was done by the Ambion Company (TX, USA) using a patented siRNA design algorithm applied to the *E. histolytica Gln6Pi* gene sequence. Design was followed by an *in silico* analysis using the *E. histolytica* genome database in order to avoid matching of the candidate siRNAs with other expressed trophozoite genes. Two 21-nt siRNAs, Si 154-Gln6Pi (5′GGACAUGCAGUAUUAGGAUTT-3′) and Si 229-Gln6Pi (5′GCUGGAGAAGUUUCAUUUATT-3′), were designed and finally purchased from Applied Biosystem (USA). A siRNA with a scrambled sequence, unable to induce degradation in any cellular mRNA, was used as control of specificity (siRNA-A:sc-37007, Santa Cruz Biotechnology, CA, USA).

### 2.4. RNA Interference Assay

 The siRNAs (154-Gln6Pi and 229-Gln6Pi) were used individually and combined at amounts of 5, 10, 20, and 40 *μ*g per culture. The siRNA-A with the scrambled sequence was used at 40 *μ*g per culture following the manufacturer's instructions. Transfection of the trophozoites was performed by soaking as previously reported [17]. In brief, 1 × 10^5^ trophozoites were grown in 6 mL TYI-S33 media in culture flasks. Once reached 50% of confluence (about 5 × 10^5^ cells), cultures were added with the siRNA, individually or mixed (5, 10, 20, and 40 *μ*g of each one), and incubated at 37°C for 16 h. After the incubation, transfected trophozoites were induced to encyst by exposure to the treatment solution (4 mM H_2_O_2_ + cations, see above) during 6 h. The cells were then extensively washed with PBS pH 7.4 and the conversion rate index (percentage of CLS formed) was determined with 0.5% sarkosyl as described. Morphological evaluation and determination of chitin were carried out by observation under light microscope and calcofluor white staining, respectively.

### 2.5. Calcofluor White Staining

After the transfection with siRNA and CLS induction, calcofluor white M2R staining was performed (Sigma-Aldrich, USA). The treated cells resistant to detergents (CLS) were washed three times with PBS. Subsequent to centrifugation, pellet samples were placed onto microscope slides and several drops of 0.05% calcofluor white M2R in distilled water were added. The sample was incubated for 10 min and the slide was observed under UV light using a fluorescence microscope.

### 2.6. Viability Assay

Viability was analyzed by determining the ability of CLS to convert fluorescein diacetate (FDA) to fluorescein. The method was previously used in the infectivity determination of *G. lamblia* cysts [20]. The viability assays were performed in the cells after the transfection with siRNAs to discard the possibility of toxic effects, as well as after the treatment with 4 mM  H_2_O_2_ and 0.5% sarkosyl. In brief, treated trophozoites were centrifuged, pelleted, and washed three times with PBS pH 7.4. Afterwards, the cells were counted and adjusted to 1 × 10^6^ mL with PBS. Samples of 100 *μ*L were treated with 1.6 *μ*L of FDA stock solution (2.5 *μ*g/*μ*L in acetone; Invitrogen, USA), at room temperature for 8 min. Percentage of viable cells was determined by counting the number of fluorescent cells under a fluorescence microscope with a BP350–460 filter.

### 2.7. RNA Extraction and RT-PCR

Total RNA was isolated from siRNA-transfected* E. histolytica* trophozoites with TRIZOL (Invitrogen, USA). The level of expression of the *Gln6Pi* gene was determined by RT-PCR using Super Script III One-Step kit (Invitrogen, USA) following the manufacturer's instructions. An amount of 900 ng of total RNA was used for cDNA production and the PCR performed with primers previously designed to target the *E. histolytica* Gln6Pi sequence (TIGR Accession number XM_648225) [3] (Table 1). Amplification of *E. histolytica *mRNA ADP-ribosylation factor was used as control of constitutive expression (ARF; accession number XM_648949) [6, 21] (Table 1). The RT-PCR products were run in 1.5% agarose gels and quantified by densitometry using the Bio-Imaging System MiniBis (Bio-Rad, USA) and the Image J program (Image Processing and Analysis in Java) after ethidium bromide staining.

### 2.8. Western Blot

The levels of Gln6Pi protein expression in extracts from trophozoites transfected with the siRNAs were determined by western blotting. A total of 15 *μ*g protein extracts prepared with protease inhibitors were run in 10% SDS-polyacrylamide gels and transferred to nitrocellulose membranes. Protein extracts from untreated trophozoites and trophozoites transfected with siRNA-A (scrambled sequence) were used as controls of basal expression. The membranes were blocked with 0.3% PBS-Tween-5% BSA solution overnight and probed with a mouse anti-human Gln6Pi polyclonal antibody (GNPDA1, Affinity Bioreagents, USA) diluted 1 : 500 overnight at 4°C. After washing with PBS-Tween, HRP-conjugated anti-mouse IgG A M (Zymed, USA) diluted 1 : 500 was incubated with the membranes during 2 h at room temperature. Antigen-antibody reactions were detected using enhanced chemiluminescence (ECL Plus Western Blotting Detection System; GE Healthcare, UK). 

## 3. Results 

### 3.1. Interference of *E. histolytica* Gln6Pi Expression with siRNAs

**Figure 1:**
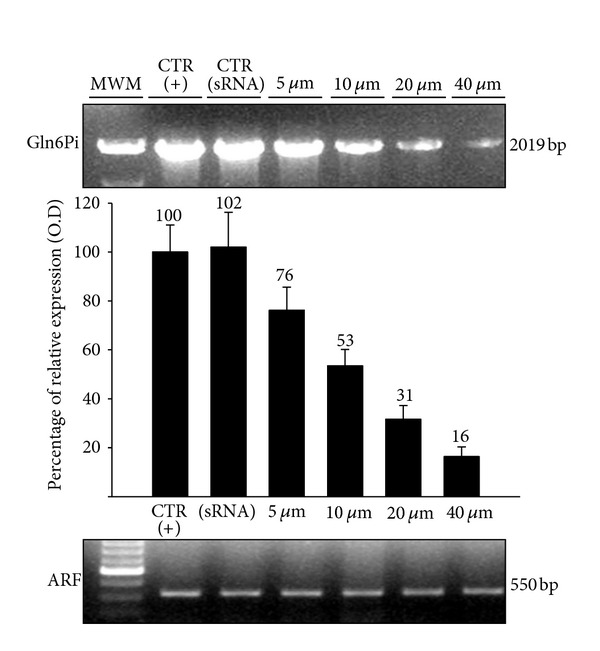
Relative expression of *E. histolytica* mRNA in trophozoites transfected with different amounts of a mix of si 154-Gln6Pi and si 229-Gln6Pi determined by RT-PCR. Trophozoites (about 5 × 10^5^ cells) were transfected with the siRNAs mix at indicated amounts by soaking during 16 h at 37°C. Afterwards, mRNA was extracted and RT-PCR performed using oligonucleotides showed in Table 1. Relative expression was determined by densitometry with respect to the constitutive expression of *E. histolytica* ADP-ribosylating factor (ARF), which was also used as loading control. CTR (+): basal expression of Gln6Pi in trophozoites; CTR (siRNA): trophozoites transfected with scrambled-sequence siRNA-A.

Treatment of *E. histolytica* trophozoites with the two siRNA probes, individually or mixed, at amounts between 5 and 40 *μ*g per 5 × 10^5^ cells did not affect the viability and replication of the parasites (data not shown). As expected, the transfected trophozoites showed decreased expression of Gln6PI RNA in RT-PCR assays when the parasites were treated with either siRNA (data not shown). However, the interference effect was greater when the parasite was treated with the mix of siRNAs. The effect was dose dependent with the mix of 5 *μ*g of each siRNA inhibiting about 24% and the mix of 40 *μ*g inhibiting almost 85% of Gln6Pi expression, when compared to the basal expression of Gln6Pi in untreated trophozoites and trophozoites transfected with the scrambled sequence, where the basal expression of Gln6Pi was similar (Figure 1). 

**Figure 2:**
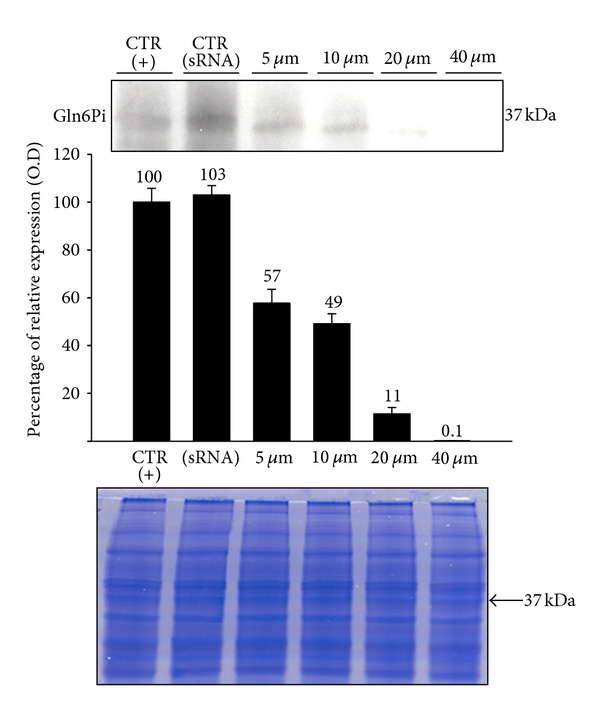
Levels of *E. histolytica* Gln6Pi protein expression in trophozoites transfected with different amounts of a mix of si 154-Gln6Pi and si 229-Gln6Pi determined by western blot. Trophozoites (about 5 × 10^5^ cells) were tranfected with siRNAs mix at indicated amounts by soaking during 16 h at 37°C. Afterwards, total extracts were prepared in the presence of protease inhibitors, run in SDS-PAGE, and transferred to nitrocellulose paper. Gln6Pi (37 kDa) was identified by using a mouse anti-human Gln6Pi polyclonal antibody and revealed using ECL. Relative expression was determined by densitometry taking basal expression of Gln6Pi in untreated trophozoites as 100 percent (CTR (+)). CTR (siRNA): trophozoites transfected with scrambled-sequence siRNA-A. The amount of protein loaded in each lane is shown in a Coomassie stained gel.

Interference with expression of Gln6Pi was also demonstrated by western blot analysis of protein levels in extracts from interfered trophozoites. As shown in Figure 2, expression of a 37 kDa band corresponding to the molecular weight expected for Gln6Pi decreased depending on the dose of siRNA used. Thus, a mix of 5 *μ*g of each siRNA inhibited Gln6Pi expression in about 47%, whereas a mix of 40 *μ*g of each siRNA blocked completely the expression of Gln6Pi when compared with the basal protein expression of the enzyme. No effect on the expression of Gln6Pi protein was observed with the scrambled sequence (Figure 2). 

### 3.2. Effect of Gln6Pi Knockdown on the Induction of CLS

Once confirmed the downregulation of Gln6Pi expression in the interfered trophozoites, the parasites were subjected to encystment induction by treatment with hydrogen peroxide plus metal dications as indicated. Addition of 4 mM H_2_O_2_ plus dications for 6 h to parasite cultures at 37°C induced differentiation of trophozoites to CLS at different rates depending on the concentration of siRNA mix used for interference (Table 2). Thus, the conversion rate (defined as the percentage of cells resistant to 0.5% sarkosyl during 10 min) decreased as siRNA concentrations increased, ranging from 27% with 5 *μ*g to 5% with 40 *μ*g of each siRNA per culture. Accordingly, viability of parasites determined by FDA staining decreased after sarkosyl treatment from 69% with 5 *μ*g to 12% with 40 *μ*g of each siRNA. As shown in Table 2, viability of trophozoites previous to the sarkosyl treatment was not critically affected by the transfection, ranging between 87, and 95%, suggesting that the transfection procedure is not toxic to the parasite. 

### 3.3. Effect of Gln6Pi Knockdown on Chitin Expression

**Figure 3:**
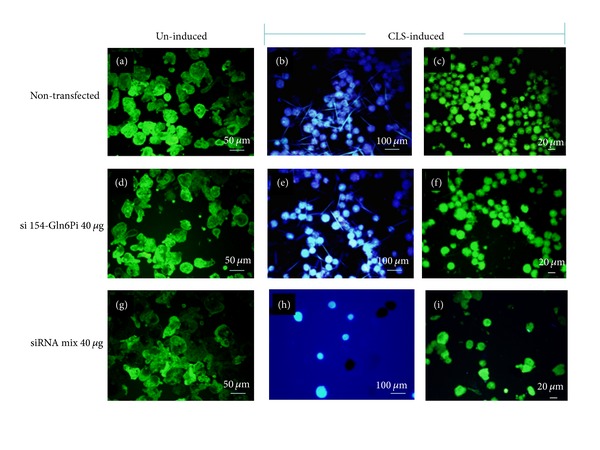
Expression of chitin and viability of Gln6Pi silenced trophozoites after CLS induction and detergent treatment. Untransfected trophozoites or transfected with 40 *μ*g of si 154-Gln6Pi or 40 *μ*g of each si 154-Gln6Pi and si 229-Gln6Pi (siRNA mix 40 ug) were induced to CLS followed by treatment with 0.5% sarkosyl. Cells were stained with FDA for viability before ((a), (d), and (g)) and after ((c), (f), and (i)) CLS induction and detergent treatment. Calcofluor white staining is shown for cells after CLS induction and detergent treatment ((b), (e), and (h)). Viable cells are observed in green and chitin-positive cells are whitish blue under UV light microscopy.

In agreement with the reduction of cell conversion rates, the increasing concentration of siRNA mix reduced the percentage of cells positive for calcofluor white staining, a disodium salt that detects specifically polysaccharides with *β* 1-3 and *β* 1-4 linkages present in cellulose and chitin (Table 2 and Figure 3). As mentioned before, transfection of *E. histolytica* trophozoites with siRNAs did not affect viability, independently of the concentration of siRNA used (Figures 3(a), 3(d), and 3(g)). 

Parasites untransfected or transfected with the single si 154-Gln6Pi at 40 *μ*g showed identical percentage of chitin positive cells (conversion rate of around 30%) after CLS induction followed by sarkosyl treatment (Figures 3(b) and 3(e)). This result is in agreement with the scarce effect of a single siRNA on Gln6Pi expression as mentioned above. In contrast, trophozoites transfected with the mix of 40 *μ*g of each siRNA showed a marked reduction in the number of chitin positive cells (Figure 3(h)), in agreement with the knockdown of Gln6Pi expression. In addition, the treated cells did not show morphological and structural features of encystment, such as refringence and multinucleation (data not shown). Noteworthy, most of the chitin positive cells were viable as determined by FDA staining (Figures 3(c), 3(f), and 3(i)). Data on viability after CLS induction, conversion rates, and calcofluor positive CLS in trophozoites transfected with different concentrations of siRNA mix are resumed in Table 2. In general, transfection with the different concentration of siRNA mix had a dose-dependent direct relationship effect on al parameters.

## 4. Discussion

Encystation is a pivotal process in the life cycle of *E. histolytica* and is indispensable for its transmission. However, the luminal stimuli triggering the process and the molecular mechanisms responsible for the stage conversion are still unknown. Recently, we have reported a reproducible *in vitro* treatment to induce cyst-like structures with most of the features of a mature cyst, including multinucleation and a chitin wall, by using a combination of hydrogen peroxide and metallic dications [3]. In the same report, we also gave evidence supporting the association between the over-expression of glucosamine-6-phosphate isomerase (Gln6Pi), the first enzyme in the theoretical route of chitin biosynthesis in amoeba, and the onset of the encystment process assayed by RT-PCR. 

In the present work, we analyzed the effect of silencing the expression of *Gln6Pi* gene on encystment of *E. histolytica* trophozoites using synthetic siRNA duplexes and the soaking method, as previously described for gene silencing in this parasite [22, 23]. As expected, transfection of *E. histolytica* trophozoites with a mixture of two siRNA duplexes during 15 h resulted in the knockdown of Gln6Pi mRNA levels in a dose-dependent manner. The mRNA levels were decreased by 85% whereas the levels of protein expression were decreased by almost 100% when parasites were soaked in a mix of 40 *μ*g of each siRNA. Our results are in agreement with the results of knockdown of *γ*-tubulin RNA and protein levels in respect of siRNA concentrations used [22]. In contrast to *Giardia lamblia*, where two *Gln6Pi* genes were identified [24], *in silico* analysis carried out by our group using the *E. histolytica* genome database suggests that this parasite may have a single gene coding for a putative Gln6Pi (data not shown). This could explain why a single interference with a mixture of two siRNAs was able to completely block the expression of the enzyme when assayed by WB. However, the exact molecular mechanism of silencing in amoeba is still unknown and waiting for demonstration, though the iRNA machinery and small RNAs are evidently present in *E. histolytica* (reviewed in [17, 18]). In this regard, several studies on *E. histolytica* G3 strain, an *in vitro* obtained strain stably silenced for the amoebapore A gene, have suggested that transcriptional and posttranscriptional mechanisms could be involved in amoeba iRNA silencing [25–28]. 

The levels of downregulation of Gln6Pi expression correlated with the decrease in the rates of *E. histolytica* encystment under induction treatment, as judged by increased susceptibility to detergent and a proportional decrease in the production of chitin-positive structures and multinucleation. Therefore, the inhibition of the Gln6Pi expression resulted in the inhibition of chitin cyst wall synthesis making the cells induced for encystment susceptible to the detergent treatment quite similar to the uninduced trophozoites. This result is in agreement with our previous proposal of Gln6Pi being the first enzyme of the pathway leading to the synthesis of *β*-(1,4)-linked N-acetylglucosamine homopolymer, the main constituent of chitin in *E. histolytica*, which in combination with other proteins such as Jessie, Jacob, and the Gal-binding lectin give place to a surface hard cover that confers resistance to harmful environmental agents, facilitating the parasite's survival and dissemination (reviewed in [8]). Thus, in combination with our previous observations [3], the present results suggest that *E. histolytica* possesses a functional metabolic pathway of chitin synthesis similar to Giardia, where the isomerization of fructose-6-phosphate to glucosamine-6-phosphate by Gln6Pi seems to be critical. However, the activities of the other four theoretical enzymes of the pathway [29] were not conducted in this study and it is a matter of further studies in our group. 

The results reported here also support the efficiency of hydrogen peroxide plus dications treatment in *in vitro* triggering the encystment process in *E. histolytica* trophozoites. The fact that stage conversion induced by hydrogen peroxide plus dications is inhibited by Gln6Pi interference, suggests that oxidative stress is able to activate, directly or indirectly, the expression of the *Gln6Pi* gene. In this regard, we have evidence of hydrogen peroxide response elements present in the promoter of *Gln6Pi* gene (data not published) that could be involved in its over-expression, in agreement with evidence of multiple genes responsive to oxidative stress having a role in cellular differentiation and many other physiological functions in eukaryotes (reviewed in [4]). However, studies on the infectivity of CLS are necessary in order to demonstrate that they are really mature cysts, and therefore, that interference of Gln6Pi will in fact inhibit the encystment *in vivo*. As mentioned above, studies on the role of the other four enzymes from the theorical pathway of chitin biosynthesis are also necessary and are actually being carried out in our laboratory.

In general, our results suggest that the encystment of *E. histolytica* is dependent on the upregulation of the Gln6Pi enzyme, which controls the onset of chitin synthesis under oxidative stress conditions. 

## Figures and Tables

**Table 1:** Oligonucleotides and siRNAs used in this work for RT-PCR and interference of Gln6Pi in *E. histolytica*, respectively.

Target gene	Primer name	Sequence (5′ to 3′)
*gln6Pi *	Gln6PiEh-F	ATGTCATCCACAAACGAAAATATTC
*gnl6Pi *	Gln6PiEh-R	CAATAGACATGGATTTATCATATC
*EhARF *	EhARF-F	GTAGGACTTGATGCTGCC
*EhARF *	EhARF-R	TCACCATTAGTTGCAC
*gln6Pi-mRNA *	siRNA 154-Gln6Pi	GGACAUGCAGUAUUAGGAUTT
*gln6Pi-mRNA *	Si 229-Gln6Pi	GCUGGAGAAGUUUCAUUUATT
—	siRNA-A:sc37007	—

Controls for constitutive expression (ARF primers) and specificity of interference (siRNA-A) are also shown.

**Table 2:** Effect of Gln6Pi knockdown by a mix of si 154-Gln6Pi and si 229-Gln6Pi siRNAs on viability, conversion rate and chitin expression of trophozoites induced to CLS.

(siRNA mix)	Interference trophozoites viability (FDA) (% ± S.D.)	Treatment solution^a^/incubation time	Conversion rate (% ± S.D.)^b^	Viability (FDA) (% ± S.D.)	Staining calcofluor white (% ± S.D.)
5 *μ*g	95 ± 3.6	4 mM/6 h	27 ± 4.4	69 ± 5	95 ± 2
10 *μ*g	91 ± 1.5	4 mM/6 h	19 ± 5.3	65 ± 4.5	90 ± 8
20 *μ*g	90 ± 2.3	4 mM/6 h	9 ± 3	36 ± 1.1	65 ± 2
40 *μ*g	87 ± 3	4 mM/6 h	5 ± 2.1	12 ± 2.8	15 ± 4.8

^a^Hydrogen peroxide 30% containing traces of several dications, see Section  2.2.

^b^Three independent experiments were done by triplicate.

Conversion rate: percentage of cells that were resistant to 0.5% sarkosyl.

FDA: fluorescein diacetate.

S.D.: standard deviation.
